# Utility of arsenic-treated bird skins for DNA extraction

**DOI:** 10.1186/1756-0500-4-197

**Published:** 2011-06-15

**Authors:** Till Töpfer, Anita Gamauf, Elisabeth Haring

**Affiliations:** 1 Biodiversity and Climate Research Centre (BiK-F), Senckenberganlage 25, 60325 Frankfurt/M., Germany; 2 Senckenberg Natural History Collections Dresden, Museum of Zoology, Königsbrücker Landstrasse 159, 01109 Dresden, Germany; 3 Museum of Natural History Vienna, 1st Zoological Department, Bird Collection, Burgring 7, 1010 Vienna, Austria; 4 University of Vienna, Department of Evolutionary Biology, Althanstrasse 14, 1090 Vienna, Austria; 5 Museum of Natural History Vienna, 1st Zoological Department, Laboratory of Molecular Systematics, Burgring 7, 1010 Vienna, Austria

## Abstract

**Background:**

Natural history museums receive a rapidly growing number of requests for tissue samples from preserved specimens for DNA-based studies. Traditionally, dried vertebrate specimens were treated with arsenic because of its toxicity and insect-repellent effect. Arsenic has negative effects on *in vivo *DNA repair enzymes and consequently may inhibit PCR performance. In bird collections, foot pad samples are often requested since the feet were not regularly treated with arsenic and because they are assumed to provide substantial amounts of DNA. However, the actual influence of arsenic on DNA analyses has never been tested.

**Findings:**

PCR success of both foot pad and body skin samples was significantly lower in arsenic-treated samples. In general, foot pads performed better than body skin samples. Moreover, PCR success depends on collection date in which younger samples yielded better results. While the addition of arsenic solution to the PCR mixture had a clear negative effect on PCR performance after the threshold of 5.4 μg/μl, such high doses of arsenic are highly unlikely to occur in dried zoological specimens.

**Conclusions:**

While lower PCR success in older samples might be due to age effects and/or DNA damage through arsenic treatment, our results show no inhibiting effect on DNA polymerase. We assume that DNA degradation proceeds more rapidly in thin tissue layers with low cell numbers that are susceptible to external abiotic influences. In contrast, in thicker parts of a specimen, such as foot pads, the outermost horny skin may act as an additional barrier. Since foot pads often performed better than body skin samples, the intention to preserve morphologically important structures of a specimen still conflicts with the aim to obtain optimal PCR success. Thus, body skin samples from recently collected specimens should be considered as alternative sources of DNA.

## Background

Currently, natural history museums all over the world receive a rapidly growing number of requests for tissue samples from preserved specimens for DNA-based examinations. The demand for extensive taxon sampling, coupled with the ease of applying molecular techniques, encourages many researchers to analyse specimens kept in museum collections. Thus, museum specimens, in parallel with blood and tissue collections, are of growing importance for molecular studies, particularly when rare or extinct species are required for phylogenetic analysis or when comparison between extant and historical populations is the focus [1-8]. However, the majority of preserved specimens have not been collected for molecular analyses and serve as valuable vouchers for various other studies. Tissue sampling results in irreversible and at least partial physical damage to the specimens [8,9], and such demand is in disagreement with the curatorial duty to preserve specimens in the best possible condition. Therefore, it is desirable to develop sampling strategies that reduce destructive effects to a minimum whilst accommodating the needs of molecular studies.

For the purpose of DNA analysis, the majority of preserved avian specimens are sampled by cutting off foot (or toe) pads or part of them [10]. As foot pads may provide other useful insight, e.g. into patterns of eco-morphological adaptations (e.g. [11]), such sampling causes substantial structural loss, especially in small birds [8]. In contrast, body skin may allow repeated invisible sampling due to its greater availability and because the feathers conceal the sampled area. The medial area where the bird has been opened during preparation is easily accessible and therefore most convenient for sampling purposes. However, foot pad samples are frequently requested because it is assumed that they provide substantial amounts of DNA. This may be considered necessary for old specimens containing DNA that is presumably degraded [10]. Moreover, unlike the remaining parts of bird skins, the feet were not always treated with arsenic.

Arsenic is commonly used to preserve dried vertebrate specimens because of its toxicity to pest insects (overview in [12]). Usually, a 10% arsenic solution is brushed on the inside of the skin during preparation and/or is distributed as a powder in the plumage or fur. Because of health concerns, the use of arsenic has been banned in many vertebrate collections [12]. In old specimens, the existing arsenic concentration may vary to an unknown extent and taxidermists did not routinely document whether they had used arsenic.

Since arsenic has a deleterious effect on *in vivo *DNA repair enzymes [13-17], it may inhibit PCR performance. Thus, as the feet were not normally treated with arsenic, foot pad samples were considered reliable sources of DNA negating the potential negative effects on PCR [10]. However, the actual influence of arsenic on PCR has never been specifically investigated.

In this study, we analyse the applicability of sampling body skin instead of foot pads in order to avoid destructive sampling of the feet, thus preserving them for subsequent studies. We compare PCR success of body skin samples with foot pad samples. Furthermore, we examine the actual potential of arsenic as a PCR inhibitor. Firstly, in a comparative approach, we investigate possible differences in PCR success between arsenic-treated and untreated samples. Secondly, we test the impact of arsenic on PCR success experimentally.

## Materials and methods

We analysed 64 individual study skins of the European Jay *Garrulus glandarius *from the ornithological collection of the Natural History Museum Vienna (Table 1). In this collection, arsenic was used for specimen preservation until 1971 after which it was replaced by Eulan (chlorphenylid). Thus we preferentially included individuals that have been collected within a narrow timescale before and after the 1971-timeline. To control for potential effects of specimen age, we included additional, particularly old specimens to check for a potential age bias in our interpretation. In order to cover a range of collection dates it was necessary to sample different subspecies of *G. glandarius*.

**Table 1:** Specimens of *Garrulus glandarius *included in this study

year	feet	skin	taxon	NMW no.	lab no.
1871	330	330	*G. g. krynicki*	3.302	Gglakry4

1892	192	192	*G. g. glandarius*	22.961	Gglagla29

1882	192	192	*G. g. glandarius*	22.960	Gglagla30

1896	192	192	*G. g. glandarius*	22.936	Gglagla31

1908	192	192	*G. g. japonicus*	83.271	Gglajap2

1913	330	330	*G. g. glandarius*	75.691	Gglagla32

1918	600	600	*G. g. glandarius*	75.713	Gglagla28

1918	600	192	*G. g. glandarius*	75.721	Gglagla33

1918	330	192	*G. g. glandarius*	75.722	Gglagla34

1919	330	330	*G. g. glandarius*	75.699	Gglagla35

1919	330	192	*G. g. glandarius*	75.700	Gglagla36

1924	330	330	*G. g. glandarius*	63.998	Gglagla37

1927	600	600	*G. g. glandarius*	84.239	Gglagla67

1929	600	192	*G. g. glandarius*	22.933	Gglagla39

1930	330	600	*G. g. glandarius*	22.935	Gglagla40

1931	600	600	*G. g. glandarius*	397	Gglagla27

1935	600	600	*G. g. glandarius*	6.165	Gglagla41

1935	600	600	*G. g. glandarius*	6.169	Gglagla42

1935	600	330	*G. g. rhodius*	83.689	Gglarho1

1941	600	192	*G. g. glandarius*	75.734	Gglagla43

1941	330	192	*G. g. fasciatus*	45.417	Gglafas1

1942	600	192	*G. g. glandarius*	75.739	Gglagla44

1942	600	600	*G. g. glandarius*	45.405	Gglagla45

1942	600	192	*G. g. glandarius*	75.737	Gglagla46

1943	600	330	*G. g. glandarius*	45.403	Gglagla47

1943	600	600	*G. g. glandarius*	45.404	Gglagla48

1943	600	192	*G. g. glandarius*	45.402	Gglagla49

1943	600	600	*G. g. glandarius*	45.401	Gglagla50

1958	330	192	*G. g. rufitergum*	93.013	Gglaruf1

1959	330	192	*G. g. glandarius*	93.540	Gglagla25

1961	600	330	*G. g. glandarius*	86.271	Gglagla26

1962	600	192	*G. g. rufitergum*	93.015	Gglaruf2

1962	330	192	*G. g. rufitergum*	93.016	Gglaruf3

1967	600	600	*G. g. graecus*	72.179	Gglagrc1

1968	600	600	*G. g. krynicki*	72.418	Gglakry3

1970	330	600	*G. g. atricapillus*	76.678	Gglaatr1

1972	600	330	*G. g. glandarius*	72.606	Gglagla17

1975	600	600	*G. g. glandarius*	73.118	Gglagla18

1981	600	192	*G. g. glandarius*	78.428	Gglagla19

1981	600	600	*G. g. glandarius*	78.427	Gglagla20

1981	600	330	*G. g. glandarius*	78.459	Gglagla21

1983	600	600	*G. g. glandarius*	78.462	Gglagla22

1983	330	330	*G. g. glandarius*	78.460	Gglagla23

1983	600	600	*G. g. glandarius*	78.493	Gglagla51

1983	600	600	*G. g. glandarius*	78.495	Gglagla52

1983	600	600	*G. g. glandarius*	78.494	Gglagla53

1983	600	600	*G. g. glandarius*	78.469	Gglagla54

1983	600	600	*G. g. glandarius*	78.467	Gglagla55

1983	600	600	*G. g. glandarius*	78.468	Gglagla56

1983	600	600	*G. g. glandarius*	78.475	Gglagla57

1983	600	600	*G. g. glandarius*	78.473	Gglagla58

1983	600	600	*G. g. glandarius*	78.474	Gglagla59

1983	600	600	*G. g. glandarius*	78.472	Gglagla60

1983	600	600	*G. g. glandarius*	78.478	Gglagla61

1983	600	600	*G. g. glandarius*	78.479	Gglagla62

1983	600	600	*G. g. glandarius*	78.477	Gglagla63

1985	600	600	*G. g. glandarius*	81.225	Gglagla64

1985	600	192	*G. g. glandarius*	81.226	Gglagla24

1987	600	600	*G. g. glandarius*	84.076	Gglagla65

1988	600	600	*G. g. glandarius*	82.614	Gglagla66

1989	600	600	*G. g. glandarius*	75.732	Gglagla38

1990	600	600	*G. g. glandarius*	85.916	Gglagla68

1990	600	330	*G. g. glandarius*	85.917	Gglagla69

1990	600	600	*G. g. glandarius*	85.915	Gglagla70

2002	-	-	*G. g. glandarius*	-	Gglagla6

2002	-	-	*G. g. glandarius*	-	Gglagla8

Rows "feet" and "skin" denote maximum amplicon lengths of foot pads and skin tissue samples, respectively. Samples Gglagla6 and Gglagla8 consist of fresh tissue material and were used as templates for PCR control and arsenic concentration experiments.

Specimens were placed on a clean sheet of paper and sampled individually. Both a foot pad and a skin sample from the bird's belly were taken from each specimen using sterile scalpels and forceps. Both paper and equipment was exchanged after sampling a specimen. To obtain skin samples, the body plumage of the ventral surface was separated. A piece of skin was cut off from an unfeathered area of the belly, preferably where the bird was opened during preparation. Foot pad samples were cut from the fleshy ventral parts of the feet and toes. We tried to compensate for the compactness of foot pads compared to the sheet-like skin patches by taking slightly larger skin pieces: the size of a foot pad sample was ~ 2 × 2 mm, integument samples measured ~ 3 × 3 mm. Extracted DNA of two fresh tissue samples (Gglagla6 and Gglagla8, Table 1) was used as a positive control.

DNA extraction was performed using the Agowa sbeadex forensic kit (Agowa GmbH, Berlin), following the standard protocol except incubation time and elution volume: Incubation of body skin samples was about 12 to 16 hours, for foot pad samples up to 24 hours depending on visible progress of tissue digestion. The final elution volume of DNA solution was 20 μl. Polymerase chain reaction of a partial sequence of the mitochondrial control region (CR) was performed in 25 μl final reaction volume containing 3.0 μl template DNA, applying the following conditions: 3 min of pre-denaturation at 94°C, followed by 35 cycles of denaturation for 30 sec at 94°C; primer annealing for 30 sec at 58°C; elongation for 40 sec at 72°C, and final elongation for 10 min at 72°C before cooling to 4°C. We used four PCR primers (Table 2). By combining the forward primer with each of the three reverse primers three PCR products of different lengths could be amplified (lengths referring to Gglagla17): CR-Cor14+/Phe-Cor- (600 bp), CR-Cor14+/CR-Cor13-/(330 bp), and CR-Cor14+/CR-Cor12- (192 bp). Amplification products were analysed by electrophoresis in 1.0% agarose gels before sequencing. If negative, individual PCRs were repeated two more times before being considered as negative. The authenticity of the respective DNA sequences was determined by comparison to *G. glandarius *reference sequences taken from [18]. All 600 bp PCR fragments were purified from agarose gels using the Qiaquick Gel Extraction Kit (Qiagen) and cloned (TOPO TA Cloning Kit, Invitrogen) prior to sequencing. Sequencing of cloned PCR products (both strands) was performed with universal M13 primers by AGOWA.

**Table 2:** PCR primers used in this study

Primer	Sequence (5'-3')	Source
CR-Cor14+	GGAGTTATCTTCCTCTTGAC	Designed for this study

Phe-Cor-	TTGACATCTTCAGTGTCATGC	[31]

CR-Cor13-	GGTGGTTTGGATAATGTAGGT	Designed for this study

CR-Cor12-	GAAACATGTCCGGCAACCAT	Designed for this study

### Influence of arsenic on PCR performance

In an additional experiment the influence of arsenic on PCR performance was tested by adding stepwise increasing concentrations of sodium arsenite (NaAsO_2_) solution to the PCR mixture (resulting final concentrations ranged from 4.6 - 6 μg/μl). We took the identical sodium arsenite as used by the NHM's taxidermists, who usually applied a ~10% arsenic solution to the skins, in order to repeat the most common specimen treatment at many natural history museums authentically. DNA of a fresh tissue sample (Gglagla8) served as template. Primers CR-Cor14+ and CR-Cor13- (330 bp) were used and PCR cycle conditions were identical to the other samples.

The data interpretation is based on multivariate statistics. As the data set is composed by non-interval-scaled and not independent variables, logistic regression with repeated measurements (Wald test) was used. Wald test is a parametric statistical test in which the maximum likelihood estimate of the parameters of interest is compared with the proposed value assuming their difference to be approximately normally distributed. The square of the difference is compared to a chi-squared distribution [19]. All calculations were done using SPSS 17.0.

## Results

In general, PCR amplification yielded clear bands of the expected fragment sizes. Sequencing of PCR products confirmed all sequences to be authentic mt CR amplicons, either identical or very similar to the published *G. glandarius *references.

### PCR success from arsenic-treated and untreated foot pad and body skin samples

**Figure 1:**
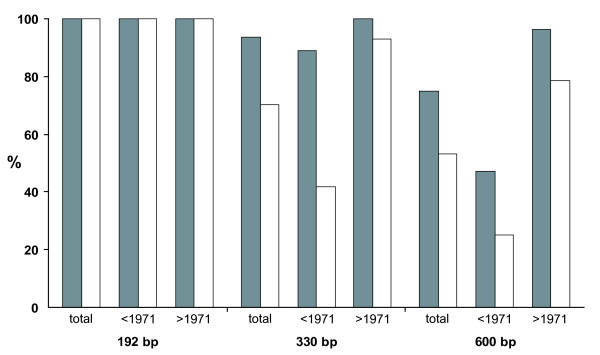
**Overall PCR success**. PCR success (in percent) of three mt CR amplicons. For each amplicon, total success and success per period (until 1971 & after 1971) is given. Grey columns - foot pads, empty columns - skin samples. Note that pre-1971 samples consist of arsenic-treated samples, post-1971 samples were untreated.

We evaluated PCR success as a general measure of usability of tissue samples considering two aspects: (1) presence/absence of PCR product in general, (2) maximum amplicon length. An overview is given in Table 3. Regarding overall PCR success (Figure 1), none of the samples was completely negative.

**Table 3:** Overall PCR success of foot pad and body skin samples

		192 bp	330 bp	600 bp
**skin**	As-treated (n = 36)	36 (100%)	19 (53%)	12 (33%)

	untreated (n = 28)	28 (100%)	26 (93%)	22 (78%)

	total (n = 64)	64 (100%)	45 (70%)	34 (53%)

**feet**	As-treated (n = 36)	36 (100%)	32 (89%)	21 (58%)

	untreated (n = 28)	28 (100%)	28 (100%)	27 (96%)

	total (n = 64)	64 (100%)	60 (94%)	48 (75%)

**skin & feet**	As-treated (n = 72)	72 (100%)	51 (71%)	33 (46%)

	untreated (n = 56)	56 (100%)	54 (96%)	49 (88%)

	total (n = 128)	128 (100%)	105 (82%)	82 (64%)

Numbers of samples and percentage with successful PCR are given per sample type and amplicon length. Arsenic-treated samples are from the pre-1971 period.

All samples irrespective of collection time and tissue type were successful for the 192 bp-amplicon. With respect to the larger amplicons (330 bp, 600 bp) skin and foot pad samples differed considerably (Wald χ^2 ^= 19.024, P < 0.001). This was also tested separately for arsenic-treated and untreated samples. In both cases foot pad samples performed better (before 1971: Wald χ^2 ^= 26.665, P < 0.001; after 1971: Wald χ^2 ^= 4.382; P = 0.036). Furthermore, general PCR success was significantly influenced by the age of the sample, in which younger samples performed better. This was tested separately for the two time periods (before 1971: Wald χ^2 ^= 6.347, P = 0.012; after 1971: Wald χ^2 ^= 14.709; P < 0.001) as well as for the whole sample (Wald χ^2 ^= 22.896, P < 0.001). Finally, the maximum amplicon length obtained depended significantly on sample age (before 1971: Wald χ^2 ^= 30.754, P < 0.001; after 1971: Wald χ^2 ^= 29.758; P < 0.001; complete sample: Wald χ^2 ^= 26.186, P < 0.001).

**Figure 2:**
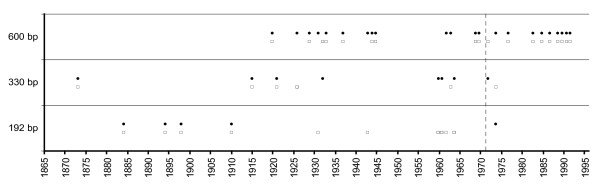
**Distribution of collection dates of successfully amplified samples**. Only the maximum amplicon lengths are indicated. Black dots: foot pads, empty squares: skin samples. Some symbols may represent several samples from the same year with the same PCR success. The dashed line indicates the 1971 timeline after which the use of arsenic ceased.

The relationship between age of sample and PCR performance can also be seen in Figure 2 and Table 3: A higher proportion of successful PCR was obtained from younger samples. For the 600 bp-amplicon this was the case with DNA from both tissue types, whereas for the 330 bp-amplicon foot pads performed only slightly better. From specimens older than 1915 the 600 bp-amplicon could not be obtained at all. However, this was also the case for several younger samples (i.e., maximum amplicon length 192 bp).

Finally, we tested the influence of arsenic treatment on PCR success for the complete sample, excluding the collection date for this calculation. Arsenic-treated samples performed significantly worse (Wald χ^2 ^= 20.400, P < 0.001). However, it should be noted that the effect of sample age cannot be excluded completely from the analysis as all samples from before 1971 had been treated with arsenic.

### PCR experiment using arsenic

**Figure 3:**
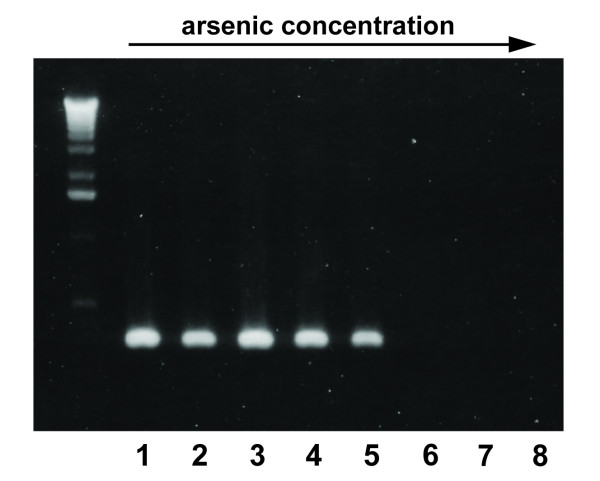
**Influence of arsenic concentration on PCR performance**. DNA template from fresh tissue sample Gglagla8. Arsenic concentrations (μg/μl): lanes 1-8: 0 - 4.6 - 4.8 - 5.0 - 5.2 - 5.4 - 6.0 - control sample (without DNA or arsenic). Signal intensity decreases with increasing arsenic concentration and breaks down from sample 6 (5.4 μg/μl).

The addition of arsenic solution of increasing concentration to the PCR mixture had a clear negative impact on PCR performance (Figure 3). However, this effect did not increase continuously with rising arsenic concentrations. Compared to the sample without arsenic (lane 1) DNA amplification proceeded without visible inhibition until a certain threshold was passed (lanes 6 to 8). This threshold was reached at a sodium arsenite concentration of 5.4 μg/μl.

## Discussion

While both foot pad and body skin samples yielded mtCR DNA sequences, the two tissue types differed in the proportion of successful PCR amplifications (Figure 1, Table 3). For both the 330 bp and the 600 bp-amplicon foot pads proved to be more successful and mostly showed somewhat stronger signal intensity on electrophoresis gels. Practically, this is not problematic, as even samples with weak gel signals yielded sufficient DNA for sequencing purposes. Nonetheless, it would be interesting to learn more about the reasons for the different performance.

### Disentangling effects of tissue type, age and arsenic treatment

One explanation for the better performance of foot pads in the PCR experiment is the assumption that DNA degradation is lower in foot pads compared to body skin. We also cannot rule out the possibility that skin samples in general contain less DNA owing to smaller cell numbers than in foot pads. However, it appears plausible that DNA degradation might proceed more rapidly in thin body skin that is more exposed to external influences such as temperature and humidity compared to thicker tissue structures of a specimen, e.g. foot pads. This might explain the lower PCR success with longer amplicons (600 bp and 330 bp, respectively) from body skin samples (Figure 1). The outermost horny skin of the foot pads may represent an additional barrier. Since moisture is an important factor promoting DNA degradation by fostering enzymatic activity [20-22], the treatment of skins during preparation (e.g., under field conditions) as well as transport and storage conditions crucially influence DNA quality. Consequently, we cannot conclude which of these factors had the greatest influence on PCR results. At least the fact that general PCR success in all samples is reciprocally proportional to amplicon size (192 bp-amplicon 100%, 330 bp-amplicon 82%, 600 bp-amplicon 64%) suggests that differences in DNA quality are crucial factors rather than solely DNA concentration.

The influence of collection date on PCR success (Figures 1, 2) is ambiguous and cannot be generalised. Although our data show a correspondence of specimen age and PCR performance, DNA degradation might proceed at different rates in each specimen depending on its individual history. Apart from the rule-of-thumb that recent samples might deliver a larger amount of non-degraded DNA that allow the generation of longer amplicons (cf. e.g. [23]) there are many individual exceptions (Figure 2). The data presented here confirms findings of other studies [24-27] that the maximum amplicon length varies individually, depending not only on a specimen's age but also on the respective collectors or taxidermists.

The actual impact of the specimens' arsenic content on PCR performance could not be determined unequivocally. Since we do not know to which extent the feet were actually treated with arsenic, we assumed an identical treatment both for skin and foot pad samples. Although statistics showed a significant effect of arsenic treatment on overall PCR success rate, it is evident that the time factor cannot be eliminated from the test, simply because the arsenic-treated samples are the older ones. Thus, lower amplification success in arsenic-treated samples might also be attributable to individual age effects and different rates of DNA degradation (Figure 1).

### PCR experiment using arsenic

The common assumption that arsenic might inhibit PCR is based on the known mutagenic effects of arsenic. Arsenic itself is unlikely to cause gene mutations directly [14,28], but can induce DNA strand breaks by mediation of reactive oxygen species in living cells [29]. However, these reports refer to living systems and are not directly comparable to *in vitro *processes. Although our PCR experiment with different tissue types suggests that arsenic has an influence on PCR success, this effect is doubtlessly superimposed by age-dependent DNA degradation. Besides potential DNA damage, arsenic could also influence PCR performance by interaction with DNA polymerase. A number of studies demonstrate arsenic to inhibit DNA transcription factors [13] and to impede *in vivo *DNA repair mechanisms by binding to polymerases [13-17]. In particular, arsenic attachment to zinc finger domains of proteins hampers normal polymerase activity [17]. Assuming that arsenic directly inhibits polymerase activity, this inhibition should be observed with any amplicon length in *in vitro *experiments. Nevertheless, this was not the case in our experiments with various museum specimens. Our PCR experiment using increasing arsenic concentrations further disproved this assumption: regarding the high concentrations necessary to inhibit PCR in our experiment (~ 5.4 μg/μl, Figure 3) we consider such high doses of arsenic to be highly unlikely to occur in dried zoological specimens like bird skins. This is corroborated by the findings of [12] who detected arsenic concentrations as low as 0.935 mg/l and 0.173 mg/l in skin and feather samples respectively. Although we did not perform particular measurements, we assume that arsenic concentration in the final DNA elution used in our comparisons of the two tissue types was much lower than in our PCR experiment: The highly DNA-specific extraction protocol is based on purification by means of a DNA-selective membrane and the discard of any other cellular compound. We therefore consider the contribution of arsenic in dried museum specimens to be negligible for a potential inhibition of DNA polymerase.

### Sampling museum specimens

A main question of this study was whether sampling of anatomically scarce components of a specimen (foot pads) could be substituted by less critical parts (body skin). In contrast to [10] we neither regard foot pad sampling as "non-destructive" nor the damage done to the feet as "negligible" in morphological respect. This is particularly important for small-sized specimens and those of rare taxa that cannot be re-obtained. When using body skin instead of cutting off foot or toe pads, care should be taken not to damage those areas where the skin is sewn in order to avoid impairing the skin's stability (in small birds the skin often remains unsewn and potential sampling areas can be reached easily). Alternatively, punch biopsy samples can be taken instead of using scalpels [30]. By doing so, there should be sufficient skin material remaining for other examinations and replication of experiments. The negligible effect of this procedure on the specimen's appearance is comparable to carefully sampling single feathers from preserved skins as recommended by [8].

In our study, the two tissue types were compared to find out whether they are equally useful regarding PCR success. The results show that, especially for old specimens, foot pad samples are the better choice. Nonetheless, for particularly valuable, small, or rare specimens the use of skin samples should be considered first. We are convinced that using body skin samples can relieve ornithological and other vertebrate collections, at least partly, of the need to cause irreversible damage to specimens. This might be a reasonable alternative for recently collected specimens.

## Conclusions

This study shows that the arsenic content in body skin samples of dried specimens has a negligible effect on DNA polymerase efficacy as inhibition occurs only at very high concentrations that are unlikely to be found in dried zoological specimens. However, potential DNA damage due to arsenic treatment cannot be ruled out, although such an effect is hard to disentangle from normal DNA degradation in old samples. Nonetheless, irrespective of arsenic content, PCR success proved to be significantly better with DNA from foot pads compared to body skin, especially for old specimens. Therefore, the conflict between the intention to preserve morphologically important structures and the need for DNA sources yielding optimal PCR success remains. In order not to compromise the future research potential of zoological specimens, both interests should be balanced carefully.

## Competing interests

The authors declare that they have no competing interests.

## Authors' contributions

TT collected samples and carried out the experiments. TT, AG and EH jointly prepared the experimental design, contributed to data interpretation and analysis and drafted the manuscript. AG contributed to sample collection and performed the statistical analysis. The authors have read and approved the final version of the manuscript.
